# Facial Deformity in an Operated Case of Bilateral Temporomandibular Joint Ankylosis

**DOI:** 10.7759/cureus.60857

**Published:** 2024-05-22

**Authors:** Anannya Mishra, Nitin Bhola, Rajanikanth K

**Affiliations:** 1 Oral and Maxillofacial Surgery, Sharad Pawar Dental College and Hospital, Datta Meghe Institute of Higher Education & Research, Wardha, IND

**Keywords:** temporomandibular joint ankylosis, coronoidectomy, retrognathia, pharyngeal airway, mandibular distraction osteogenesis

## Abstract

Temporomandibular joint (TMJ) ankylosis results in malocclusion, poor feeding, difficulty in maintaining oral hygiene, and facial esthetic deformity. The basic surgical objectives in the treatment of TMJ ankylosis are to establish joint movement, prevent relapse, and achieve normal growth and development. Here, we present an operated case ofsurgical correction of mandibular hypoplasia; however, the patient came back after three years due to unsatisfactory results and underwent bilateral coronoidectomy and gap arthroplasty. Bones were osteotomized at the LeFort I level and the maxillary segment was down-fractured and mobilized to bring into occlusion with the mandible. In the present case, the lower pharyngeal airway changed from 5 mm pre-treatment to 10 mm post-treatment, and the facial angle was changed from 73 to 84 post-treatment. Assessment of the pharyngeal airway is done with a high suspicion of obstructive sleep apnea and facial deformity is mandatory in the management of TMJ ankylosis.

## Introduction

The temporomandibular joint (TMJ) is a finely balanced structure with a high degree of anatomic precision. TMJ ankylosis can be defined as an "inability to open the mouth due to either a fibrous or bony union between the head of the condyle and the glenoid fossa" [1]. Mandibular condyle injury is the most common cause of TMJ ankylosis, which is a significant issue in Asian nations. Among others, infection, delivery using forceps, trauma, and rheumatoid arthritis are some less frequent causes [2].

In situations of TMJ ankylosis, mandibular retrognathia is increasingly being corrected by mandibular distraction osteogenesis (MDO) [3]. Orthognathic surgery (OGS) and distraction osteogenesis (DO) have been widely recommended for a variety of craniofacial abnormalities. The best course of correction is still up for debate, particularly when it comes to clinical indications and patient-related outcomes, including stability, long-term growth influence, sociopsychological ramifications, and quality of life [4]. When treating TMJ ankylosis, there are three main surgical goals. These are to achieve normal growth and development, establish joint movement, and avert relapse [5]. Here, we present an operated case of surgical correction of mandibular hypoplasia; however, the patient came back after three years due to unsatisfactory results and underwent bilateral coronoidectomy and gap arthroplasty. Bones were osteotomized at the LeFort I level and the maxillary segment was down-fractured and mobilized to bring into occlusion with the mandible.

## Case presentation

A 24-year-old female patient reported to the Department of Orthodontics with a chief complaint of difficulty in opening her mouth and irregular face shape. The patient complained of the inability to close lips for three years. The patient was fine three years back when she noticed an inability to close her lips. The patient had a history of inability to close lips, reduced mouth opening, and difficulty in mastication and deglutition since childhood. There was no history of difficulty in speech or any ear, nose, & throat infections. The patient visited the hospital 13 years back where she was operated on for bilateral TMJ ankylosis for which release of ankylotic mass and reconstruction with costochondral graft was done on the right side under general anesthesia, which was uneventful (details were not present with the patient).

**Figure 1:**
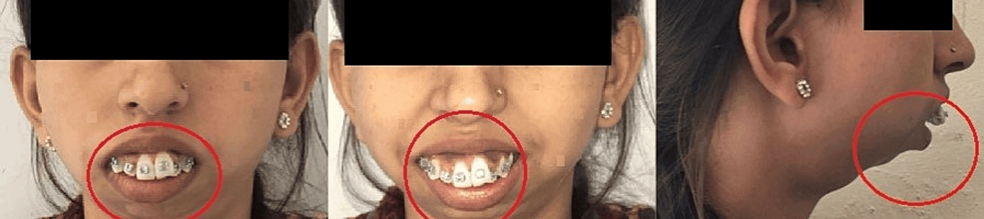
Pre-treatment extra-oral picture Image A shows the pre-treatment frontal photograph. Image B shows the pre-treatment frontal smiling photograph. Image C shows the pre-treatment lateral photograph.

The patient then visited the hospital where her orthodontic treatment was started (Figure 1). The patient was then admitted to the oral surgery ward of Acharya Vinoba Bhave Hospital in 2019 where the patient's consent prior to corrective surgery was taken. The procedure of surgery was explained to the patient and surgical correction of mandibular hypoplasia by mandibular osteotomy, bilateral distraction osteogenesis, and LeFort I osteotomy with maxillary superior impaction were done. Following this, distraction osteogenesis (bilateral distraction = 14 mm; dancing distraction on the left side = 4 mm) was done.

**Figure 2:**
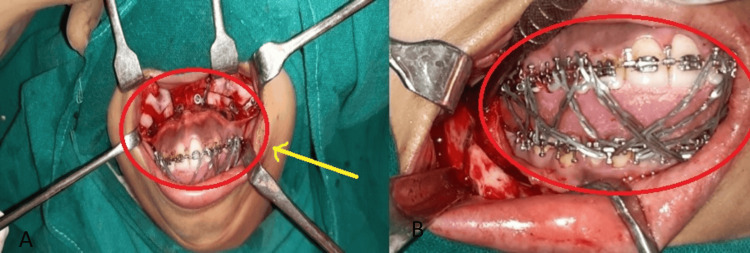
Surgical removal of distractor and advancement genioplasty in an operated case of mandibular hypoplasia (2020) Image A shows the placement of bilateral distractors. Image B shows the maxillo-mandibular fixation.

**Figure 3:**
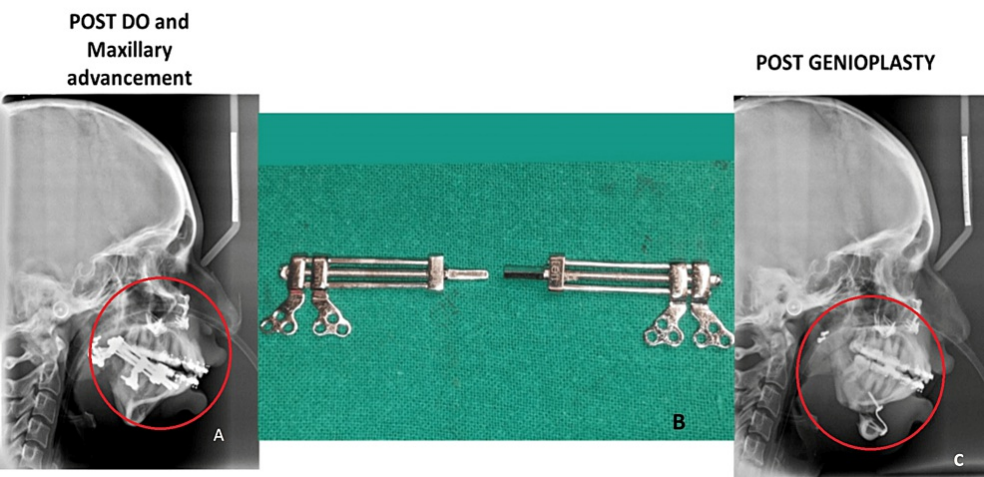
Post distraction osteogenesis (DO) and maxillary advancement A: Circle A denotes findings post distraction osteogenesis and maxillary advancement. B: Circle B denotes bilateral distractors. C: Circle C denotes findings post genioplasty.

The patient was again operated on in 2020 where surgical removal of distractor and advancement genioplasty in an operated case of mandibular hypoplasia was done (Figure 2). Figure 3 shows a sequential lateral cephalogram after distraction osteogenesis (DO) and maxillary advancement, and post-genioplasty image.

**Figure 4:**
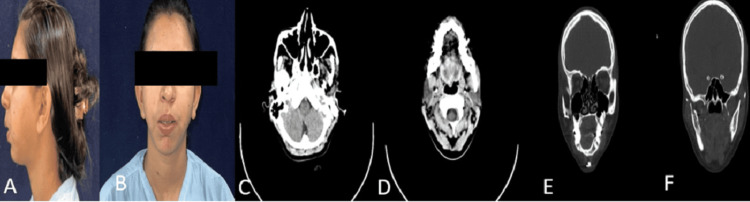
(A and B) Extra-oral profile (2023); (C and D) CT scan; (E and F) CT scan (coronal cuts)

The patient reported for further management after three years as she was not satisfied with her facial appearance and had difficulty closing her mouth due to her incompetent lips (Figures 4A, 4B). The chief complaint was the inability to join lips and facial asymmetry for approximately three years. The patient was advised an orthopantomogram (OPG), lateral cephalogram, and computed tomography scan (Figures 4C-4F).

**Figure 5:**
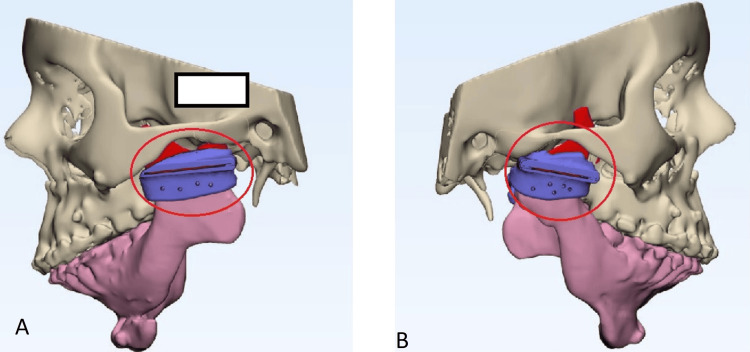
Cutting guide placement for osteotomy cuts and placement of the condylar component The red circle in images A & B signifies the virtual component guide for planning osteotomy cuts before the surgery.

Treatment was planned with the objective to improve quality of life and to correct facial deformity (Figure 5). Surgery was advised to move apart osteotomized bone and for neo-bone regeneration between the two separated bony ends due to a deficient mandible, reduced airway, and vertical maxillary excess. The goal of the orthodontic surgical technique was to segment the maxilla to broaden, narrow, level, or improve arch symmetry, as well as to surgically realign the entire dentoalveolar segment superiorly, inferiorly, anteriorly, and posteriorly. It was planned in two stages: in stage I, LeFort superior impaction, and in stage II, removal of distractor and genioplasty. The surgical algorithm was bilateral TMJ replacement using a patient-specific implant, leading to rotation of the entire mandibular complex forward and anticlockwise rotation of the maxilla (re-osteotomy at LeFort I level with the anterior nasal spine (ANS) as the point of rotation).

**Figure 6:**
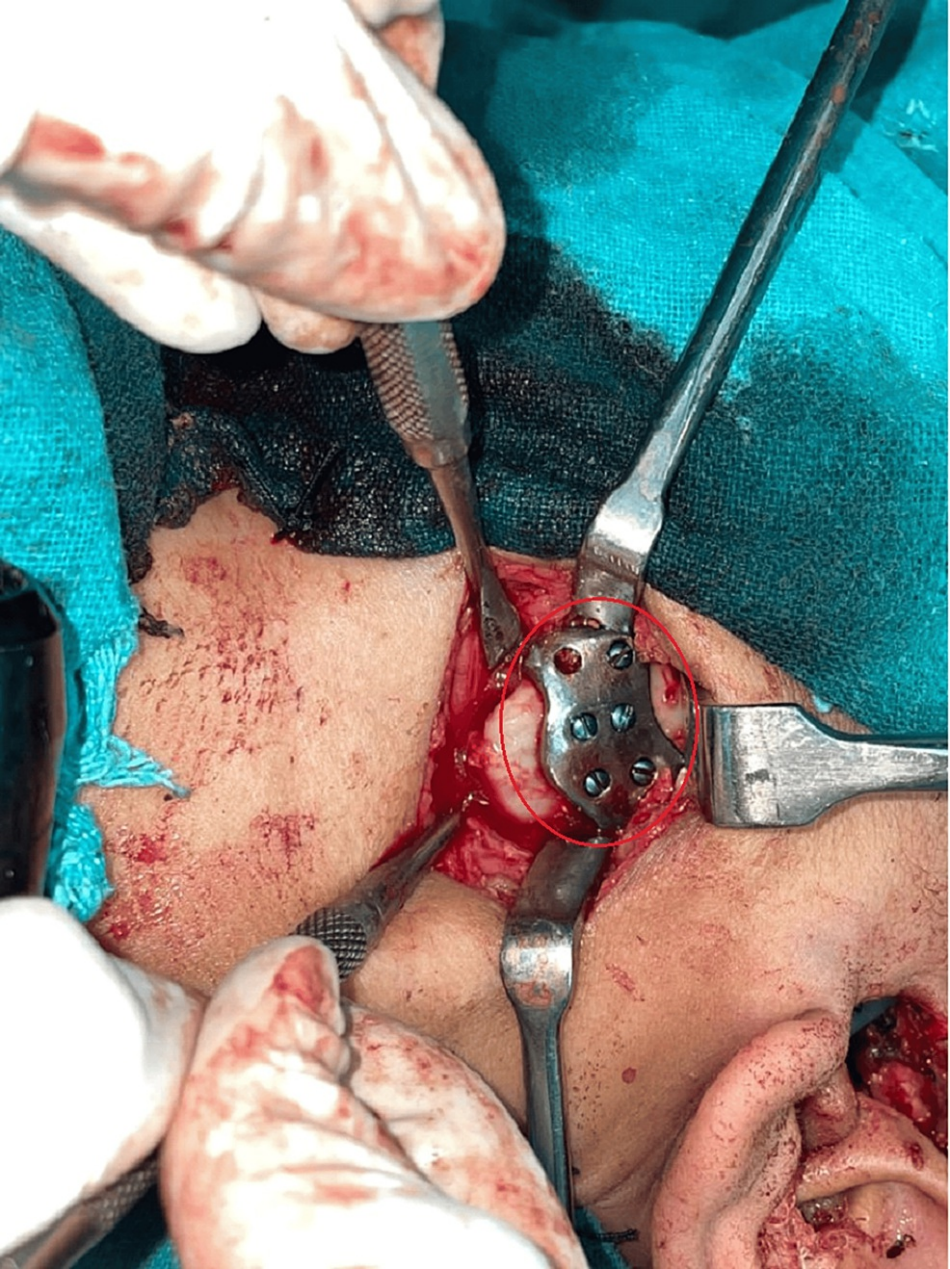
Placement of the condylar component The red circle signifies the placement of the condylar component.

Standard markings were done for bilateral pre-auricular and peri-angular incisions. The incision was deepened through the skin and subcutaneous tissue and reached the zygomatic arch and angle of the mandible, respectively. The bilateral TMJ coronoid process was exposed and released. A bilateral coronoidectomy was performed and gap arthroplasty was done. Reconstruction was done with patient-specific titanium condyle and ultra-high molecular polyethylene glenoid fossa unit over both sides. A maxillary vestibular incision was given from the 16 to 26 region. The incision was deepened through the mucosa, submucosa, muscle, and periosteum. The full thickness of the mucoperiosteal flap was elevated preserving the infraorbital nerve bilaterally. Dissection was done from ANS exposing nasal lining mucosa and zygomaticomaxillary buttress. Osteotomy cuts were marked at the LeFort I level. Bones were osteotomized at the LeFort I level, and the maxillary segment was down-fractured and mobilized to bring into occlusion with the mandible. Intermaxillary fixation (IMF) screws were secured over 17, 27, 37, and 47 regions. Occlusion was brought up in Angle's class I relation and a splint was placed and fixed with E-chain. The condyle and coronoid process were removed from both sides and were used as bone grafts and were fixated using titanium plates and screws. A pressure dressing was given over the bilateral preauricular region, upper lip, and periangular region (Figure 6).

**Figure 7:**
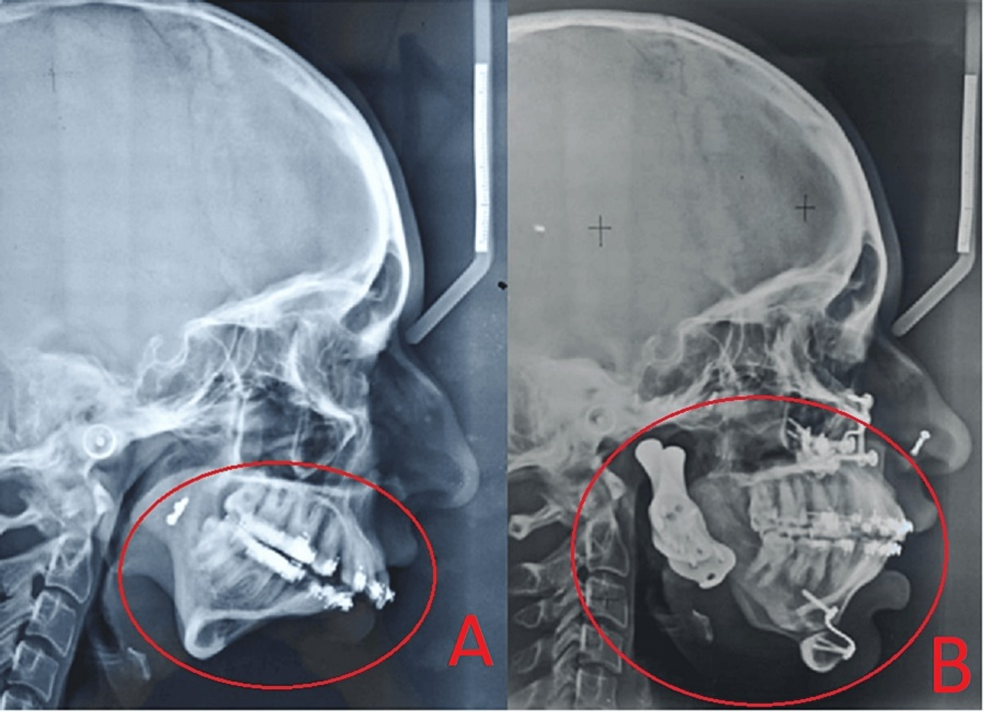
Comparison of pre and current cephalometric measurements Image A shows the cephalogram taken before the total joint replacement. Image B shows the cephalogram taken after total joint replacement.

**Figure 8:**
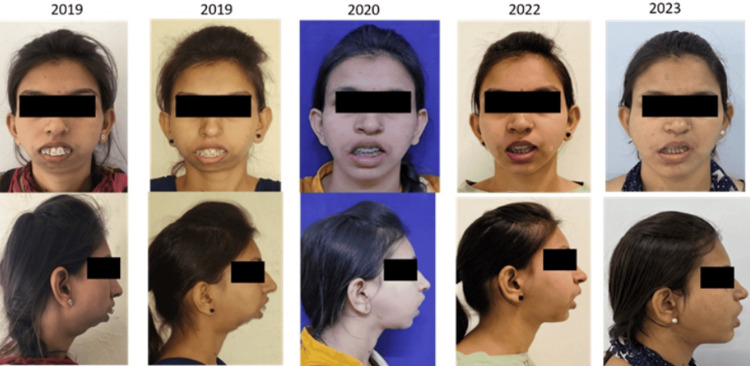
Sequential extra-oral photographs from 2019 to 2023

Figure 7 shows a comparison of pre and current cephalometric measurements. Figure 8 shows sequential extra-oral photographs from 2019 to 2023. Mouth-opening exercises four times daily for 20-30 minutes using ice cream sticks/bite blocks were advised at discharge. The patient was followed up after one week.

## Discussion

TMJ ankylosis causes difficulty in mastication as well as swallowing food and speaking difficulties, mandibular deformity, developmental impairment, and poor oral hygiene that leads to dental caries and periodontal disease. Teeth eruption and location are adversely affected by malformed alveolar processes. The afflicted patient's facial feature is frequently referred to as "bird profile." The mandible is clearly retruded and lacks the chin; the nasolabial angle is bigger than normal; the cervical mental angle is obtuse; and the lower face is significantly shortened. The chin is noticeably shifted to the side that is afflicted, resulting in an asymmetrical face. The lower lip is seen to be incompetent, wedged beneath the maxillary front teeth [6]. To explain the pathophysiology of TMJ ankylosis, numerous theories have been proposed. Every theory that has been put forth centers on joint trauma followed by recovery, which results in ankylosis. This is only the case for a small percentage of patients, as the majority do not experience ankylosis following TMJ damage, whether or not they seek therapy [2].

Due to its distinct anatomy and relationship to several key tissues, most notably the facial nerve and the auriculotemporal nerve, the TMJ has presented challenges to otolaryngologists, oral and maxillofacial surgeons, and other clinicians over the years [1]. A treatment plan for TMJ ankylosis is described, following the guidelines set forth by Kaban et al. [7,8]. This plan includes the following: (1) aggressive resection; (2) ipsilateral coronoidectomy; (3) contralateral coronoidectomy when necessary; (4) temporalis fascia or cartilage lining the TMJ; (5) reconstruction of the ramus with a costochondral graft; (6) rigid fixation; and (7) early mobilization and aggressive physiotherapy [7,8]. Exercises that strengthen and relax the masticatory muscles are part of physiotherapy. The goals of physical therapy are decreasing edema, causing scar tissue to relax and stretch, expanding the joint range of motion, and strengthening the masticatory muscles [9].

Computed tomography of the ankylosed joint in the axial, coronal, and sagittal planes is required for preoperative imaging. Determining the extent of the bone mass medially assists in evaluating closeness to the significant vascular structures in the infratemporal fossa when contrast is utilized [10]. Preoperative evaluation by computed tomography images was done in the present case. Gap arthroplasty, which involves removing the bony mass without using any inter-positional material, joint reconstruction, which involves removing the bony mass and reconstructing it using bone grafts or joint prosthesis, and inter-positional arthroplasty, which involves removing the bony mass and using either biological or non-biological material, are the fundamental surgical techniques for treating ankylosis [11].

The present patient started her orthodontic treatment and underwent surgical correction of mandibular hypoplasia by mandibular osteotomy, bilateral distraction osteogenesis, and LeFort I osteotomy with maxillary superior impaction done in 2019, following which distraction was done. Later, in 2020, surgical removal of the distractor and advancement genioplasty was done in an operated case of mandibular hypoplasia. However, the patient again visited the department after three years. The treatment was planned with the objective to improve the quality of life and to correct facial deformity. Surgery was advised to move apart osteotomized bone and for neo-bone regeneration between the two separated bony ends due to a deficient mandible, reduced airway, and vertical maxillary excess. Orthognathic surgical procedure was planned for surgical repositioning of the entire dentoalveolar segment of the maxilla superiorly, inferiorly, anteriorly, and posteriorly, while simultaneously segmentalizing it to widen, narrow, level, or improve arch symmetry. A bilateral coronoidectomy was performed and gap arthroplasty was done. The surgery was planned in two stages: stage I, Le-fort superior impaction; stage II, removal of distractor and genioplasty.

In a different instance, as documented by Al-Nuumani et al. [3], a nine-year-old boy underwent surgical management that included ipsilateral coronoidectomy, distractors removal, TMJ ankylosis release, and condyle reconstruction using an inter-positional fat graft and a costochondral rib graft.

Lengthening of the bone and distraction of soft tissue are two of DO's primary benefits. Simultaneously, the surrounding soft tissues are "recruited" or "stretched" due to the bone's slow and deliberate movement [12]. Limited mouth opening and re-ankylosis are the most frequent side effects following ankylosis surgery. There have also been cases of Frey's syndrome, anterior open bite, and temporary facial nerve paresis [13]. In this case, there were no postoperative complications reported.

The mandible's growth is impacted by TMJ ankylosis, which causes severe deformities of the face. Retrognathia is a crucial clinical characteristic of long-term TMJ ankylosis, and if it is paired with an inability to open the mouth, it can seriously restrict breathing [14]. In the present case, the facial angle was altered from 73 to 84 post-treatment, and the lower pharyngeal airway was increased from 5 mm pre-treatment to 10 mm post-treatment.

Treatment for TMJ ankylosis should begin as soon as the illness is identified. Restoring mandibular mobility and promoting further growth should be the goals of early treatment to lessen the likelihood of future facial asymmetry. The small ramus condyle unit may limit mid-facial growth, cause considerable malocclusion, and cause unilateral mandibular retrusion to emerge later [13]. TMJ ankylosis can only be treated surgically. If adequate function is to be restored, early surgical repair of the ankylosed joint is extremely desired [6]. The surgical approach used is determined by the age at which ankylosis first appears, the severity of the condition, whether it affects one or both sides, and any related facial deformities [15].

## Conclusions

In this instance, TMJ ankylosis led to malocclusion, difficulty in feeding, trouble keeping teeth clean, and a distortion in the appearance of the face. Both the restricted pharyngeal airway space and the dental difficulties can be resolved by surgically releasing the ankylosis. To treat TMJ ankylosis, pharyngeal airway assessment must be performed with a high suspicion of obstructive sleep apnea and facial deformities.
